# Identification of a Depolymerase Specific for K64-Serotype *Klebsiella pneumoniae:* Potential Applications in Capsular Typing and Treatment

**DOI:** 10.3390/antibiotics10020144

**Published:** 2021-02-01

**Authors:** Jiayin Li, Yueying Sheng, Ruijing Ma, Mengsha Xu, Fuli Liu, Rong Qin, Mingxi Zhu, Xianchao Zhu, Ping He

**Affiliations:** 1 Department of Medical Immunology and Microbiology, Shanghai Jiao Tong University School of Medicine, Shanghai 200025, China; jiayinlee@sjtu.edu.cn (J.L.); xums5812@enzemed.com (M.X.); 2111807117@zjut.edu.cn (F.L.); ashinqr@sjtu.edu.cn (R.Q.); simpsons@sjtu.edu.cn (M.Z.); 2 College of Medical Technology, Shanghai University of Medicine & Health Sciences, Shanghai 201318, China; shengyy@sumhs.edu.cn; 3 Shanghai Ruizhou Biotech Co. Ltd., Pudong New District, Shanghai 201203, China; rjma@ruizhoubio.com

**Keywords:** *Klebsiella pneumoniae*, bacteriophage, capsular depolymerase, capsular typing, antivirulence

## Abstract

Carbapenem-resistant *Klebsiella pneumoniae* (CRKP), one of the major nosocomial pathogens, is increasingly becoming a serious threat to global public health. There is an urgent need to develop effective therapeutic and preventive approaches to combat the pathogen. Here, we identified and characterized a novel capsule depolymerase (K64-ORF41) derived from *Klebsiella* phage SH-KP152410, which showed specific activities for *K. pneumoniae* K64-serotype. We showed that this depolymerase could be used in the identification of K64 serotypes based on the capsular typing, and the results agreed well with those from the conventional serotyping method using antisera. From this study, we also identified K64 mutant strains, which showed typing discrepancy between *wzi*-sequencing based genotyping and depolymerase-based or antiserum-based typing methods. Further investigation indicated that the mutant strain has an insertion sequence (IS) in *wcaJ*, which led to the alteration of the capsular serotype structure. We further demonstrated that K64-ORF41 depolymerase could sensitize the bacteria to serum or neutrophil killing by degrading the capsular polysaccharide. In summary, the identified K64 depolymerase proves to be an accurate and reliable tool for capsular typing, which will facilitate the preventive intervention such as vaccine development. In addition, the polymerase may represent a potential and promising therapeutic biologics against CRKP-K64 infections.

## 1. Introduction

*Klebsiella pneumoniae*, a Gram-negative, rod-shaped, and encapsulated opportunistic pathogen, causes a wide range of nosocomial infections, including septicemia, pneumonia, urinary tract infections (UTI) and liver abscesses [1]. Various antimicrobials have been used to treat *K. pneumoniae* infections, which has led to a significant rise of antibiotic resistant strains. In the past decades, the infections by multiple-drug resistant (MDR) *K. pneumoniae* have increasingly emerged with high mortality rates, particularly in hospitals [2,3]. Among the MDR strains, Carbapenem-resistant *K. pneumoniae* (CRKP) has posed a significant challenge to the global public health due to lack of the available antibiotics [4]. In recent years, immune therapy and phage-based therapy have gradually drawn more attentions from the scientific and industrial communities [5,6].

Capsular polysaccharide (CPS) has been used as an effective vaccine target for manypathogenic bacteria, such as *Streptococcus pneumoniae* and *Neisseria meningitidis*. *K. pneumoniae* is classified into at least 78 capsular serotypes based on the serological reaction of CPS with specific antisera [7,8]. It is important to determine capsular types of clinical isolates prior to application of any specific medical treatment or future development of CPS-based vaccines [9]. The capsular types of *Klebsiella* can be identified by a variety of methods. The traditional serological method in determining capsular types was established in 1926 [10]. However, due to the limited source and high cost of antisera, tedious experimental procedures, and the large number of serological cross-reactions, few laboratories were capable of using the serological method to determine the capsular types of *K. pneumoniae* strains [9,11]. Apart from the serological method, new genotyping methods have been developed for the capsular typing based on the single loci of the capsule polysaccharide (*cps*) synthesis region, such as the conserved *wzi* [12], *wzc* [13], and *wzy* gene [14]. The current epidemiological investigation of the capsular distribution is mostly based on *wzi* genotyping. In recent years, bacteriophages and phage-derived capsule depolymerases have been suggested as a valuable approach for capsular typing [15,16], while the capsule depolymerases show higher specificities than their phages [10,16,17].

As one of the main virulence factors, CPS protects the bacteria through evading from the detection of the host immune system [18], including opsonophagocytosis and serum killing [19,20], and acts as a potent barrier against antimicrobials [21,22]. Recently, the phage-derived capsular depolymerase has emerged as an alternative agent to treat *K. pneumoniae* infections [23]. Depolymerase can degrade the CPS on the bacterial surface, which makes bacteria readily recognized and destroyed by the host immune system [24]. It has also been reported that specific depolymerases can reduce the virulence of Gram-negative enteric bacteria, such as *Escherichia coli*, *K. pneumoniae,* and *Acinetobacter baumannii* [25], and enhance the killing effect of serum and neutrophils in vitro [10,15]. In addition, depolymerases can stably maintain their activity also in vivo and have no toxic effect on mice [26]. In the previously published studies, a K1-specific depolymerase was shown to significantly improve the survival rate of mice infected with a *K. pneumoniae* K1 strain [27,28]. The combination of the phage-derived depolymerase with other therapeutic agents has thus brought up a new therapeutic strategy for the treatment of MDR *K. pneumoniae* infections.

Recently, K64 *K. pneumoniae* has been identified as a common pathogen among CRKP strains as detected and reported in Brazil [29], Singapore [30], Taiwan [31], the United States, and Europe [32]. According to previous epidemiology studies, *K. pneumoniae* K64 serotype is one of the most prevalent types in CRKP infections in China [33]. In this study, we identified a phage-derived depolymerase (K64-ORF41), which can specifically degrade the bacterial capsule of K64 serotype *K. pneumoniae*. We also investigated its potential applications in the capsular typing and anti-CRKP infections. Our results showed that this depolymerase can be used as a reliable and easy method for K64 capsular typing, as well as an alternative therapeutic agent for treating K64 *K. pneumoniae*.

## 2. Results

### 2.1. Capsular Genotyping of K. pneumoniae Strains

*K. pneumoniae* strain 2410 is a CRKP strain, which is resistant to multiple antibiotics, including cefotaxime (CTX), meropenem (MEM), imipenem (IPM), amikacin (AMK), cefuroxime (CXM), gentamicin (GEN), cefazolin (CZO), and piperacillin (PIP). Based on the *wzi* sequencing data, *K. pneumoniae* American Type Culture Collection (ATCC) 13,882, *K. pneumoniae* strain 2410 and strain Y8 belonged to the KL64 genotype (K-types based on the K-locus arrangement is called KL series). The antibiotic sensitivity and capsular genotypes of the rest 64 CRKP clinical strains have been reported in our previous study [34].

### 2.2. Serological Typing of K. pneumoniae Strains

We used the traditional serological methods, namely the Quellung assay and the agglutination test, to further confirm the genotyping results for these K64 clinical strains in this study. The K64-antiserum was used for these two serological typing methods. The Quellung assay and the agglutination test were performed using *K. pneumoniae* ATCC 13,882 and 66 CRKP clinical strains. The results showed that *K. pneumoniae* ATCC 13,882 and 39 of 47(83%) genotyped KL64 clinical strains reacted positively with the K64-antisera (Figure S1), and these strains were defined as KL64^+^/K64^+^ strains. Nineteen non-KL64 strains and eight genotyped KL64 strains (strain 6082, 6199, 6483, 6821, 6938, 6938, 6990, and 8206) showed negative reactions with the K64-antisera (Table 1). We defined these eight KL64 strains as KL64^+^/K64^−^ strains.

### 2.3. Isolation and Morphology of Phage SH-KP152410

**Figure 1:**
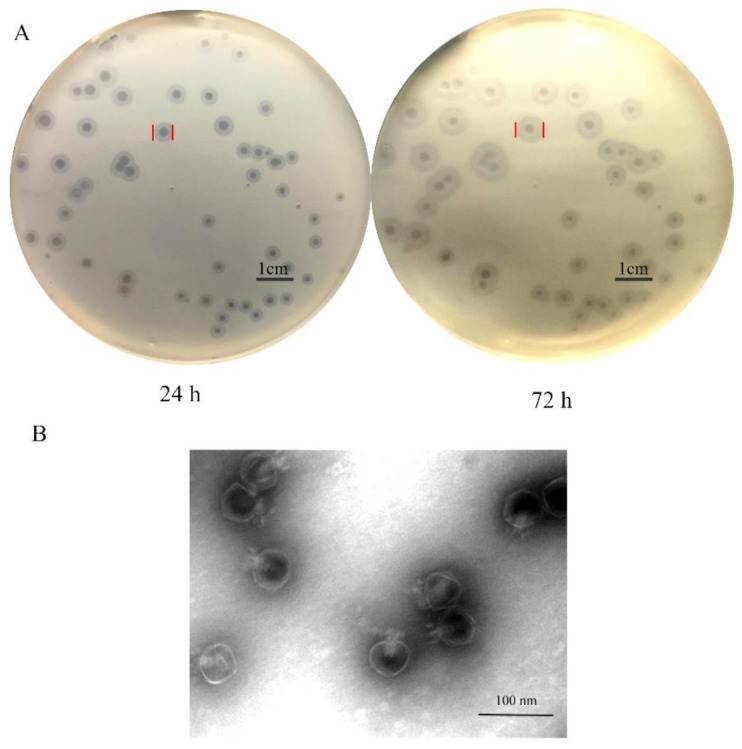
Plaques and morphological analysis of *K pneumoniae* phage SH-KP152410. (**A**) *K. pneumoniae* strain 2410 was infected with phage SH-KP152410 and the mixture was poured on the 0.75% agar and then incubated on the 1.5% agar plates at 37 °C. Plaques were observed in soft agar at 24 h and 72 h after infection. (**B**) Transmission electron micrographs of the *K. pneumoniae* phage SH-KP152410. Scale bar: 50 nm.

The phage SH-KP152410 was isolated successfully from hospital sewage samples using *K. pneumoniae* strain 2410 as the host bacterium. When added to the plate containing *K. pneumoniae* strain 2410, the isolated phage showed small, round, clear plaques of 2–3 mm in diameter with translucent halos on the double-layer agar plate (Figure 1A). The size of the halos increased over the course of 72 h (Figure 1A), which suggested the production of a polysaccharide depolymerase. From the transmission electron microscopy (TEM), we observed that the phage possessed an isometrically hexagonal head of 50 nm and a tail of 20 nm in diameter. Based on these morphological characteristics, the phage is classified to be a member of the *Podoviridae* family (Figure 1B).

### 2.4. Microbiological Characteristics and Lytic Spectrum of the SH-KP152410

Previous studies showed that the halo implied the presence of a polysaccharide depolymerase, which is a capsule-degrading enzyme of phage [35]. Hence, we deduced that infection by this phage may be related with bacterial capsular type. To prove this hypothesis, lytic spectrum of the phage was determined by spot test using 67 *K. pneumoniae* strains belonging to different capsular genotypes (Table 1). The results showed that 19 non-KL64 strains were not infected by phage SH-KP152410 (Table 1). Among the 48 KL64 genotype strains, halos surrounding the phage plaques were observed on 40 KL64^+^/K64^+^ bacterial lawns, whereas eight KL64^+^/K64^−^ strains developed neither plaques nor halos (Table 1). The results indicated that the phage SH-KP152410 had a high specificity towards the serotype K64 strains.

### 2.5. Genomic Analysis of the Phage SH-KP152410

**Figure 2:**
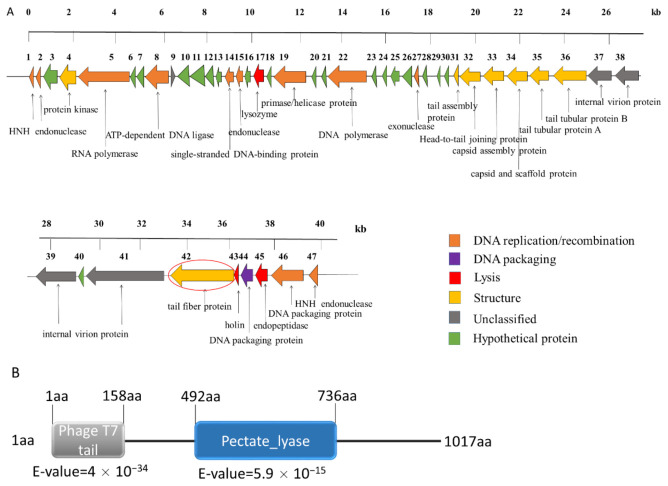
Genomic analysis of the phage SH-KP152410 and in silico analysis of the depolymerase of this phage. (**A**) Linear genome map of the phage SH-KP152410. Different colored arrows represent 47 predicted open reading frames (ORFs) with distinct functions. Orange for phage DNA replication and recombination; purple for DNA packaging; red for host lysis; yellow for structure; gray for unclassified proteins and green for hypothetical proteins. *ORF41,* the gene encoding the tail fiber protein was predicted to be a putative polysaccharide depolymerase and circled in red. (**B**) Bioinformatic analysis of the putative polysaccharide depolymerase of the phage SH-KP152410. Using BLASTp and HHpred, the result characterizes a 1017aa protein with two conserved domains: phage T7 tail fiber (residue 1–158) and pectate lyase domain (residue 492–736).

The whole-genome sequencing showed that the phage had a linear double-stranded genome of 40,945 bps, with a GC content of 52.35%. The genome of the phage SH-KP152410 was predicted to contain 46 open reading frames (ORFs). The average length of these ORFs was 776.87 bp. The tape measure protein, which is responsible for the tail assembly in the *Myoviridae* and *Siphoviridae* families, was not discovered in the genome of the phage SH-KP152410. Combined with the results of the phage morphology, the data indicated that SH-KP152410 belongs to the *Podoviridae* family. The phage-encoded proteins can be divided into six categories: DNA replication/recombination/modification protein (13 proteins), DNA packaging protein (two proteins), host lysis protein (three proteins), structure protein (eight proteins), unknown protein (four proteins), and hypothetical protein (16 proteins) (Figure 2A).

### 2.6. Prediction and Expression of the Putative Capsule Depolymerase

Phage depolymerases, often found in the tail spike or tail fiber, can degrade bacterial capsular polysaccharides into oligosaccharide units during infection [36]. The gene *ORF41* (3054 bp in length), coding for the putative tail fiber protein, was suspected to encode a polysaccharide depolymerase. The protein coded by *ORF41* was predicted to contain a conserved N-terminal phage_T7 domain and a central pectate_lyase domain with the β-helix structure after the analysis using Protein Blast (BLASTp) and HHpred (Figure 2B).

**Figure 3:**
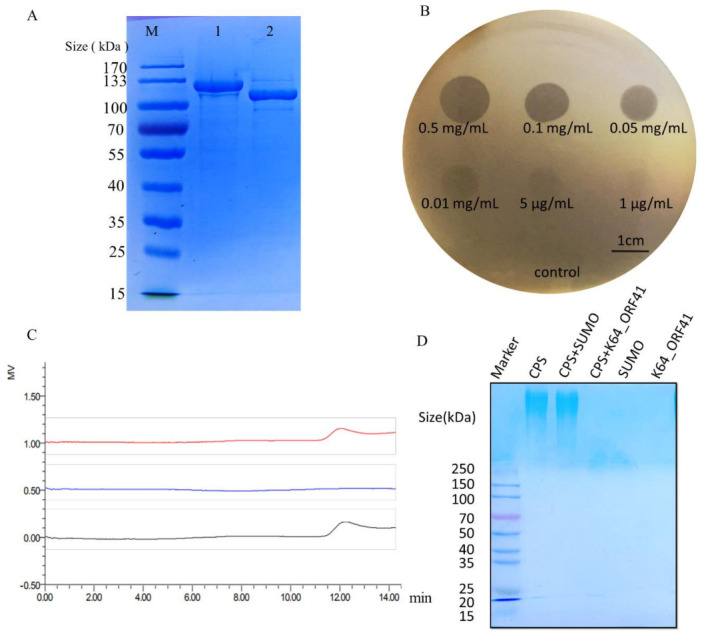
Expression, purification, and depolymerase activity evaluation of recombinant K64-ORF41. (**A**) The purified proteins were separated on 10% SDS-PAGE followed by Coomassie blue staining. Lane M, protein marker; lane 1, purified K64-ORF41-SUMO fusion protein; lane 2, K64-ORF41 without SUMO tag. (**B**) The enzymatic activity of K64-ORF41 was tested by the spot test using different concentrations on a lawn of *K. pneumoniae* strain 2410. SUMO protein was served as a negative control. (**C**) Size exclusion chromatography (SEC)-HPLC analysis of capsular polysaccharide (CPS) treated with K64-ORF41. Black line, purified untreated CPS; green line, CPS treated with 10 μg/mL K64-ORF41 at 37 °C for 30 min; red line, CPS incubated with 10 μg/mL SUMO protein at 37 °C for 30 min. (**D**) Alcian blue staining of CPS treated with the K64-ORF41. CPS was treated with or without K64-ORF41 and SUMO at 25 °C for 30 min and stained with Alcian blue. In each panel, lane 1: marker; lane 2: purified untreated CPS; line 3: CPS treated with 10 μg/mL SUMO; line 4: treated with 10 μg/mL K64-ORF41; line 5: SUMO; line 6: K64-ORF41.

To ascertain whether the protein has the depolymerase activity, the coding sequence of this gene was cloned into a pSUMO3 vector. The recombinant tagged protein was expressed and purified. The tag was cleaved with the SUMO protease and removed through the use of the Ni-NTA column. The purified protein, named K64-ORF41, migrated as a single band with a size of 112 kDa on 10% SDS-PAGE gel (Figure 3A).

### 2.7. Assessment of the Recombinant Depolymerase Activity

To assess the depolymerase activity of K64-ORF41, the spot assay was carried out and halo zones were detected on the lawn of the *K. pneumoniae* strain 2410 by using a series of dilutions of recombinant protein ranging from 0.5 mg/mL to 1 μg/mL (Figure 3B). The CPS of *K. pneumoniae* strain 2410 was extracted and purified to further confirm the depolymerization activity of K64-ORF41. As shown from the size exclusion chromatography (SEC)-HPLC in Figure 3C, the untreated CPS sample showed a single peak with a retention time from 11 to 13 min. After incubation with K64-ORF41 at 37 °C for 30 min, the peak of CPS was disappeared from 11 to 13 min, demonstrating that CPS was degraded by K64-ORF41 (Figure 3C). The capsule degrading property of K64-ORF41 was also validated by Alcian blue staining. Results of the SDS-PAGE gel revealed that the CPS of *K. pneumoniae* strain 2410 was apparently degraded by K64-ORF41, as compared with CPS alone. These results indicated that K64-ORF41 can degrade CPS from *K. pneumoniae* strain 2410 efficiently.

### 2.8. Application of K64-ORF41 for K64 Capsular Typing

The spectrum of K64-ORF41 was tested against 67 *K. pneumoniae* strains by the spot assay. As expected, halo zones were observed on the 40 KL64^+^/K64^+^ strains, but not observed on any of the 8 KL64^+^/K64^−^ strains and 19 non-KL64 strains (Table 1). The data were consistent with the results of serotyping using K64-antisera, which suggested that K64-ORF41 can be used to specifically identify *K. pneumoniae* K64 serotype.

### 2.9. ISs Occur in the cps Region of K. pneumoniae

**Figure 4:**
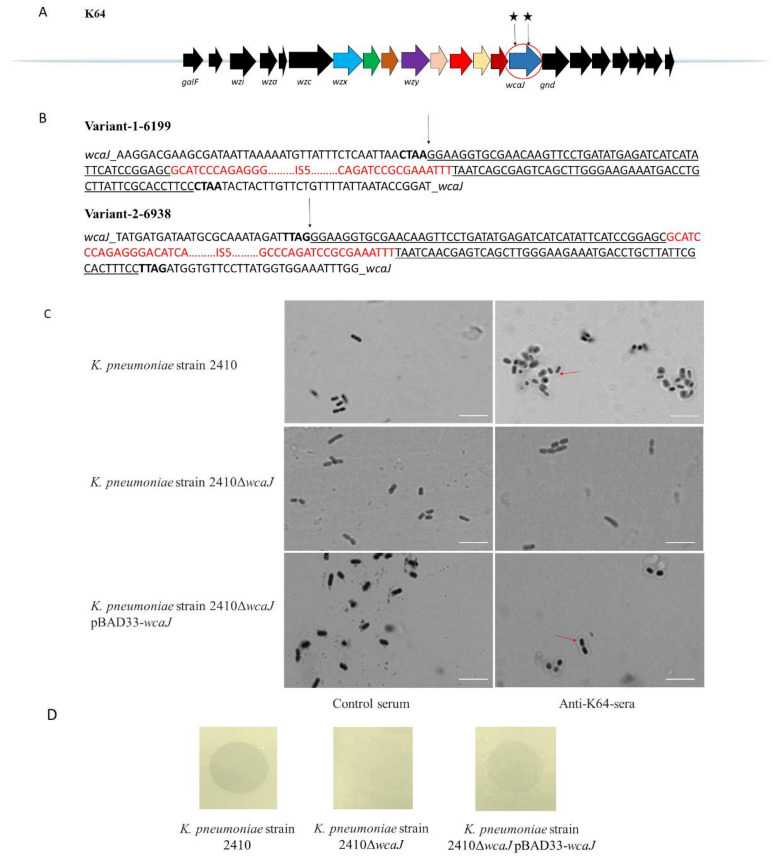
*cps*-locus variants in *K pneumoniae* clinical isolates and Quellung reaction and spot-assay test of the *wcaJ* mutant strains. (**A**) Schematic representation of the *cps* gene clusters in *K. pneumoniae* strain 6199. Open reading frames (ORFs) were shown as arrows and conserved genes were shown in black. Other gene contents were marked in different colors. The gene with insertion sequence (IS) insertions were circled in red. Blue pentacle indicated an IS*5* insertion containing variant. (**B**) Nucleotide sequence for IS*Kpn26* insertions disrupting the *wcaJ* open reading frame within the *cps* gene clusters in *K. pneumoniae* strain 6483 and 6938. Small black arrows indicated the beginning of the IS*5* sequence. Direct repeats generated by IS*5* were represented in bold. IS*5* imperfect inverted repeats were shown by underlined and IS*5* coding region were shown in red. (**C**) Quellung reaction of *K. pneumoniae* strain 2410, *K. pneumoniae* strain 2410Δ*wcaJ*, *K. pneumoniae* strain 2410Δ*wcaJ*-pBAD33-*wcaJ*, *K. pneumoniae* strain 6199-pBAD33-*wcaJ,* and *K. pneumoniae* strain 6821-pBAD33-*wcaJ*. Strains were incubated with K64-antisera or control-serum. The *wcaJ* mutant did not show the capsular swelling under the light microscope and the result was reversed upon complementation. Scale bar, 1 µm. (**D**) Spot tests of *K. pneumoniae* strain 2410, *K. pneumoniae* strain 2410Δ*wcaJ* and *K. pneumoniae* strain 2410Δ*wcaJ*-pBAD33-*wcaJ.* K64-ORF41 were spotted on the bacterial lawns. SUMO was used as a negative control.

The result that eight KL64^+^/K64^−^ strains have no reactivity with either K64-antisera or K64-ORF41, suggested that these strains might have gene mutations or interruptions among the CPS synthesis region. To investigate whether there is the discrepancy in the *cps* locus of KL64^+^/K64^−^ strains compared with the ATCC 13,882 strain, the genome of *K. pneumoniae* strain 6199 (KL64^+^/K64^−^) was sequenced and its *cps* region was analyzed. Through the bioinformatic analysis, we found that the *cps* locus of strain 6199 contains an insertion sequence (IS) within the *wcaJ* gene (Figure 4A). The *wcaJ* region of the other seven KL64^+^/K64^−^ strains were also sequenced, and the identical IS element, IS*Kpn26* (IS*5* family) was also found (Table S1). However, the IS*Kpn26* disruption was located at the different positions of the *wcaJ* gene in several isolates, such as *K. pneumoniae* strain 6483 and 6938 (Figure 4B). These results suggested that the IS*Kpn26* disruption might completely inactivate the WcaJ, an initial glycosyltransferase responsible for the first step of the capsule biosynthesis [37], leading to the change of the capsular polysaccharide structure. We further conducted the *wcaJ* deleted mutant strain to confirm our above hypothesis. Compared with *K. pneumoniae* strain 2410, 2410Δ*wcaJ* showed negative reactivity with both K64-antisera and K64-ORF41, which could be reversed by complementation with *wcaJ* (Table S2). Besides, two of these eight KL64^+^/K64^−^ strains, *K. pneumoniae* strain 6199 and *K. pneumoniae* strain 6821 were chosen to be complemented by the expression of *wcaJ.* As we expected, complemented mutants were observed to have the positive reaction with both K64-antisera and K64-ORF41 (Table S2). These results demonstrated that the IS*Kpn26* insertion in *wcaJ* resulted in the disruption of the capsular polysaccharide synthesis in serotype K64 *Klebsiella.*

### 2.10. Characterization of the Depolymerase Activity and Stability at Various pH Values and Temperatures

**Figure 5:**
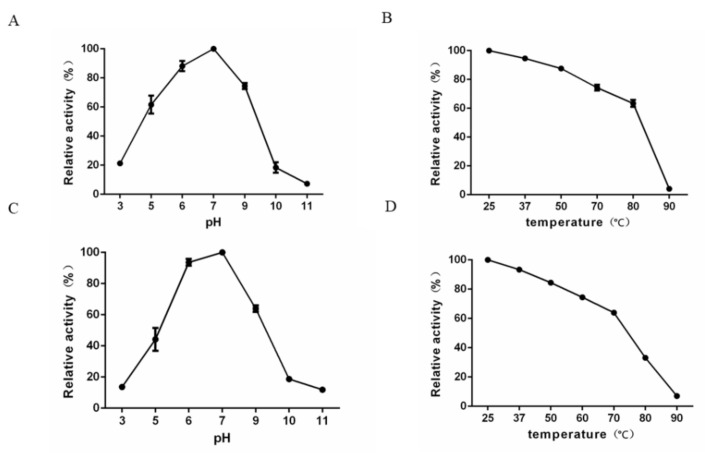
(**A**) Effect of pH on the enzymatic activity of K64-ORF41 at 25 °C. (**B**) Effect of different temperatures on the enzymatic activity of K64-ORF41 at pH 7.0. (**C**) Stability of enzyme activity under different pH values. K64-ORF41 was pre-incubated alone in different pH values for 30 min, and the residual enzyme activity was measured at 25 °C. (**D**) Stability of enzyme activity under different temperatures. K64-ORF41 was pre-incubated without substrate at various temperatures for 30 min, and the residual enzyme activity was measured at pH 7.0. The results are expressed as the relative activity by comparing with the values under optimal conditions. Data are expressed as means ± SD (*n* = 3).

The enzymatic activity of K64-ORF41 at various pH values and temperature was studied by a glucose assay using the DNS reagent. The degradation activity was determined by the measurement of reducing sugars after the digestion with the depolymerase. The optimal enzyme activity was observed at pH 7.0 and 25 °C after the incubation for 0.5 h. Relative enzymatic activity was expressed as a percentage of the optimal enzyme activity value (Figure 5). The results showed that K64-ORF41 maintained a high activity from pH 5.0 to pH 9.0, with relative activities ranging from 61.6 ± 0.98% to 100.0 ± 0.32% (Figure 5A). With respect to temperature, K64-ORF41 exhibited good activity over the range of temperature from 25 °C to 80 °C, with the relative activity from 100.0 ± 0.42% to 63.4 ± 0.54% (Figure 5B). As the temperature increased to 80 °C, the enzyme activity began to decrease gradually (Figure 5B). When K64-ORF41 was pre-incubated at pH 5.0 or pH 9.0, its relative enzymatic activity was 44.2 ± 0.89% or 64.0 ± 0.24%, respectively (Figure 5C). When pre-incubated at 80 °C for 30 min, the enzymatic activity of K64-ORF41 displayed a substantial reduction to 33.1 ± 0.42% (Figure 5D). These data demonstrated that K64-ORF41 has moderate thermostability up to 80 °C and can maintain a high activity under a wide range of pH values (5 to 9).

### 2.11. Serum Sensitivity of Depolymerase-Treated Bacteria

**Figure 6:**
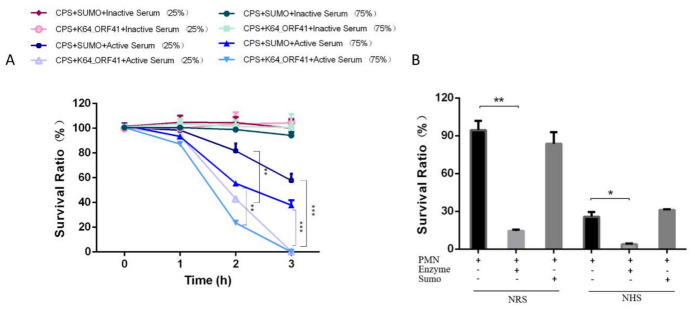
Effect of the K64-ORF41 on bacterial susceptibility to serum killing and neutrophil killing. (**A**) *K. pneumoniae* strain Y8 pretreated with K64-ORF41 were incubated with 75% or 25% normal human serum (NHS) or heat-inactivated serum for 1 h, 2 h, and 3 h at 37 °C, and SUMO protein was served as control. (**B**) *K. pneumoniae* strain Y8 was cultured with human neutrophils with or without the enzyme (K64-ORF41) as indicated. Neutrophils were pre-mixed with 5% normal rabbit serum (NRS) or 5% normal human serum (NHS). SUMO protein was served as control. The survival ratio of bacteria was determined based on viable counts with respect to the initial inoculum. Results were represented by the survival ratio from three separate experiments (mean ± SD). Significance was determined by Student’s *t* test (*, *p* < 0.05; **, *p* < 0.01; ***, *p* < 0.001).

As a major virulence factor, CPS of *K. pneumoniae* protects the bacteria from the host immune system, in particular, the bactericidal effect of serum. To investigate the anti-virulence effect of the K64 depolymerase, the serum killing assay was tested on *K. pneumoniae* strain Y8, a carbapenem-resistant hypervirulent strain (CR-hvKP) [38]. The survival of enzyme-treated bacteria incubated with active serum significantly decreased in a time-dependent manner. When the bacteria were incubated with the heat-inactivated serum, the number of surviving bacteria was almost the same as the control group (Figure 6A). After incubation with the serum for 2 h and 3 h, the viable counts of enzyme-treated bacteria were significantly decreased as compared with the control group (Figure 6A). Thus, serum complement played an important role in the serum killing assays and K64-ORF41 could significantly improve the bactericidal activity of serum against K64 CR-hvKP.

### 2.12. Neutrophil Killing of Enzyme-Treated Bacteria

We also evaluated the ability of K64-ORF41 to assist killing of *K. pneumoniae* by neutrophils. The neutrophil mediated killing assay for *K. pneumoniae* strain Y8, in the presence or absence of the enzyme treatment, was tested with the active normal human serum (NHS) or normal rabbit serum (NRS). The results showed the survival ratio of *K. pneumoniae* strain Y8 for the pretreated group was significantly decreased, compared with the untreated group (Figure 6B). Taken together, these data demonstrated that the depolymerase treatment can substantially enhance the susceptibility of the bacteria to the neutrophil killing.

## 3. Discussion

In recent years, the high prevalence of CRKP has been deemed as an urgent threat to public health [39,40]. Furthermore, carbapenem-resistant hypervirulent (hypermucoviscous) *K. pneumoniae* (CR-hvKP) isolates have been increasingly detected worldwide, especially in China. The emergence of the strains with high virulence and antibiotic resistance causes serious and untreatable invasive infections [31]. Thus, alternative methods are urgently needed for controlling and preventing the outbreak of *K. pneumoniae* infections [39,40].

Recently, the phage-derived capsular depolymerase was emerged as a promising agent to treat *K. pneumoniae* infections. So far, 24 types of polysaccharide depolymerases encoded by *Klebsiella* phage have been identified and demonstrated to have depolymerization activity against the distinct CPS of *K. pneumoniae* [41]. Of these, K56, KN1, KN3, and KN4 capsule depolymerases have been demonstrated to be efficient for capsular typing in epidemiological studies [16]. In this study, we isolated a novel K64 capsule depolymerase (K64-ORF41) and proposed the potential application of this enzyme in K64 capsular typing for the first time.

Here, we compared the depolymerase-typing with the *wzi* genotyping and traditional serological typing for *K. pneumoniae.* Based on *wzi* genotyping, 67 strains were grouped into nine distinct capsular types (highly prevalent types of CRKP infections in China) [10], including KL64 (48), KL19 (4), KL47 (3), KL60 (2), KL61 (2), KL54 (2), KL1 (2), KL2 (2), KL24 (2). Among 48 KL64 strains, 40 strains had positive reactions with K64-antisera (KL64^+^/K64^+^ strains), whereas eight were negative (KL64^+^/K64^−^ strains), consistent with the results of depolymerase-typing (Table 1). We speculated that some mutations might occur in the *cps* synthesis region (except *wzi*) of eight KL64^+^/K64^−^ strains, resulting in changes of capsular polysaccharide structure [16]. Sequencing analysis results showed there were IS*Kpn26* insertions in the *wcaJ* gene of eight KL64^+^/K64^−^ strains. As an initial glycosyltransferase, WcaJ is responsible for initiating capsule biosynthesis of strains in many capsular types [37]. IS insertion might disrupt the synthesis of capsular polysaccharide, leading to the inability of the depolymerase and K64-antisera to react with CPS of the KL64^+^/K64^−^ strains. Several studies have also reported that the IS interrupts the *wcaJ* gene in the clinical CRKP isolates, resulting in the emergence of capsule mutant strains [42,43], which should be taken into account in the design of CPS-based vaccines. To further reveal the roles of IS insertion in *wcaJ*, this gene deleted mutant was constructed. *K. pneumoniae* strain 2410-Δ*wcaJ* showed the same negative reactions with K64-antiserum and K64-ORF41 as the eight KL64^+^/K64^−^ strains, which could be recovered by complementation with *wcaJ*. Thus, mutations or interruptions in capsule biosynthesis genes disrupting the capsular polysaccharide structure would not be characterized by *wzi* genotyping. In contrast, the depolymerase typing could overcome this issue. This method proved to be a reliable method for capsular typing.

In a previously published study, Yi-Jiun Pan and his colleagues first reported a K64 depolymerase, K64dep, from the myovirus K64-1. According to the BLASTp result, K64-ORF41 exhibited limited sequence similarity with K64dep (65% query coverage and 47% identity) [10]. Sequence alignment showed that K64dep did not have a phage T7 tail fiber domain (residues 1–158) and shared 5.8% identity with K64-ORF41 at the N-terminus. The discrepancy at the N-terminal regions might be due to their origins from phages of a different family. In contrast, K64-ORF41 had a high degree of similarity with the K64dep in the central domain, which determines the enzyme activity on the capsule.

To assess the anti-virulence effect of K64-ORF41, we tested the effect of the depolymerase on the bacterial susceptibility to serum complement-mediated killing or neutrophil killing, especially against *K. pneumoniae* strain Y8, a CR-hvKP isolate highly resistant to serum and neutrophils killing [38]. In the serum-killing assay, our results showed that the survival rate of depolymerase-pretreated bacteria incubated with 75% or 25% serum was significantly decreased compared with the untreated bacteria (Figure 6A). In addition, the neutrophil killing experiment showed that the depolymerase could efficiently promote the killing of bacteria by neutrophils under the opsonophagocytosis of serum (Figure 6B). These results suggested that K64-ORF41 could be explored as a potential and promising antivirulence agent for the treatment of K64 CR-hvKP infections.

## 4. Materials and Methods

### 4.1. Bacterial Strain Isolation and Identification

A total of 67 *K. pneumoniae* strains were used in this study. The reference strain (ATCC 13882) for K64 *K. pneumoniae* was purchased from the American Type Culture Collection (ATCC) in Manassas of USA. Two strains were collected in 2015 from Huashan Hospital affiliated with Fudan University. The *K. pneumoniae* strain 2410 was used for the phage isolation and its antimicrobial susceptibility was evaluated by the Kirby–Bauer (K–B) method. *K. pneumoniae* strain Y8, a carbapenem-resistant hypervirulent (CR-hvKP) strain [38], was used for the serum assay and the neutrophil killing assay. The capsular genotypes of these 3 strains (ATCC 13,882, 2410, Y8) were determined by *wzi* sequencing according to the method of Brisse [12]. The rest 64 CRKP clinical isolates were isolated from Renji Hospital in Shanghai, China in 2019, and the capsular genotypes of these strains using the *wzi* sequencing method were reported in a previous study of our lab [34]. All clinical isolates were identified by the VITEK 2 Compact system (BioMérieux, Marcy l’Etoile, France). These clinical isolates were cultivated in Luria–Bertani broth (LB, Sangon Biotech, Shanghai, China) at 37 °C and were stored at −80 °C in glycerol (1:1 [*v*/*v*]).

### 4.2. Bacteriophage Isolation

Phage was isolated from the sewage samples, which were obtained from Ruijin Hospital, affiliated with Shanghai Jiaotong University. *K. pneumoniae* strain 2410 was co-cultured with untreated sewage in LB broth at 37 °C overnight. After centrifugation, the supernatant containing the phages was filtered with a 0.22 μm filter (Millex-GP Filter Unit; Millipore, Billerica, MA, USA) and spotted onto LB plates overlaid with *K. pneumoniae* strain 2410 for phage plaque detection. The phage titer was also determined by using an agar overlay method [44]. The lytic phage was then purified in our lab and designated as SH-KP152410. The purified phage was amplified and stored in the SM buffer (8 mM MgCl2, 100 mM NaCl, 50 mM Tris-HCl pH 7.5) at 4 °C.

### 4.3. Transmission Electron Microscopy

The phage sample was dropped onto copper grids with carbon-coated Formvar films, and air-dried for 30 min. Samples were negatively stained with phosphotungstic acid (2% *w*/*v*) and observed under a Hitachi transmission electron microscope H-9500 (Tokyo, Japan).

### 4.4. Phage Activity Spectrum

The host range of SH-KP152410 was screened by the spot test against 67 *K. pneumoniae* strains. Briefly, LB agar was overlaid with 0.75% LB agar inoculated with the log-phase bacterial cultures of *K. pneumoniae*. After a few minutes of drying, 5 μL of purified phage suspension (10^8^ PFU/mL) was spotted onto the bacterial lawn at 37 °C overnight to allow plaques to develop.

### 4.5. Genomic DNA Sequencing and Annotation

Phage genomic DNA was extracted using the standard protocol as previously described [45]. Briefly, the purified phage was treated with 1 μg/mL DNase I and RNase A (Sigma-Aldrich, St. Louis., MO, USA) at 37 °C for 1 h to remove host nucleic acids. Subsequently, the mixture was heated at 80 °C for 15 min and then added with 25 mmol/mL ethylenediaminetetraacetic acid (EDTA; Thermo Scientific, Waltham, MA, USA). Finally, the phage DNA was extracted using a commercial kit (the Qiagen Lambda Kit; Qiagen, Valencia, CA, USA). The genomic DNA was then fragmented by the ABclonal DNA Library Preparation Kit and sequenced by the Illumina HiSeq 3000 platform yielding 150 paired-end reads (Illumina, San Diego, CA, USA). The raw sequencing data was assembled using SOAPdenovo2 software [46]. Open reading frames (ORFs) were further predicted using GeneMark and the putative functions of the proteins encoded by ORFs were annotated by NCBI BLASTP [47]. The HHpred (http://toolkit.tuebingen.mpg. de/hhpred) program was used to predict the depolymerase domain [48]. The whole genome sequence of the SH-KP152410 is deposited in GenBank with the accession number (MG835568.1).

### 4.6. Cloning, Expression, and Purification of the Recombinant Depolymerase

The *ORF41* gene was amplified from the purified phage SH-KP152410 by PCR using primers K64-*ORF41*-F (5′-CAGCAGCAGACGGGAGGATCCATGGACCAAGATACTAAAACAATC-3′) and K64-*ORF41*-R (5′-CTCGAGTGCGGCCGCAAGCTTTTATGCGTTCAGGTACACCC-3′). The 3054 bp PCR amplification product was cloned into the-pSUMO3 expression vector (LifeSensors, Philadelphia, PA, USA) with an N-terminal 6xHis tag via the *BamH* I and *Hind* III sites (New England Biolabs). The recombinant plasmid was verified by DNA sequencing and transformed into the *E. coli* BL21 strain (DE3). Subsequently, the recombinant ORF41 protein, hereafter referred to as K64-ORF41, was expressed using 0.1 mM isopropyl β-d-1-thiogalactopyranoside (IPTG, Sangon Biotech, Shanghai, China) at 16 °C overnight, purified by an Ni-NTA column (GE Healthcare, Pittsburgh, PA, USA). The protein purity was analyzed using sodium dodecyl sulfate-polyacrylamide gel electrophoresis (SDS-PAGE). Finally, SUMO protease (LifeSensors, Philadelphia, PA, USA) was added to remove the His-tagged SUMO protein, and the cleaved mixture was passed through the Ni-NTA column. The purified depolymerase protein was stored at −80 °C.

### 4.7. Depolymerase Activity by the Single-Spot Assay

The depolymerase activity of K64-ORF41 was qualitatively assessed by the single-spot assay. Briefly, 400 µL of *K. pneumoniae* strain 2410 in the log-phase was mixed with 3.6 mL of 0.75% soft agar and then the mixture was poured onto 1.5% LB agar to form a bacterial lawn. Subsequently, 5 µL of the diluted enzyme was spotted on the plate and incubated at 37 °C overnight. The enzymatic activity of K64-ORF41 was monitored for the formation of translucent halo zones. In addition, the sensitivity of other 66 *K. pneumoniae* strains to K64-ORF41 (10 µg/mL) was also determined by the single-spot assay.

### 4.8. Purification of the Capsular Polysaccharide (CPS)

*K. pneumoniae* strain 2410 was cultured in the flesh Tryptone Soy Broth (TSB) medium (Sangon Biotech, Shanghai, China) overnight and incubated without stirring at 37 °C for 5 days. The purification of CPS was performed according to the method of Bales et al. [49]. Firstly, 60 μL of formaldehyde (36.5% solution) was added to each 10 mL of culture, and the mixture was cultured at 100 rpm for 1 h. Subsequently, 1 M NaOH was added to the culture solution with agitation at room temperature for 3 h and then centrifuged at 4 °C for 1 h at 16,800× *g* (Beckman, JA-25.50, San Antonio, TX, USA). The supernatant was then mixed with trichloroacetic acid (TCA, 20% *w*/*v*) to precipitate protein and nucleic acids followed by centrifugation at 16,800× *g* for 1 h at 4 °C and collection of the clear solution. Moreover, 1.5 volumes of cold ethanol (96%) was then added to the solution to precipitate the CPS at −20 °C for 24 h. The precipitate was collected by the centrifugation at 4 °C at 16,800× *g* for 1 h, and resuspended with 5 mL of ddH_2_O. The crude CPS mixture was dialyzed against 1 L of ddH_2_O by using a 12–14 kDa MWCO membrane (Thermo Scientific, Waltham, MA, USA) at 4 °C for 24 h. Finally, the dialyzed solution was lyophilized overnight and the freeze-dried powder was dissolved in ddH_2_O to 2.5 mg/mL.

### 4.9. Cleavage of K64 Capsular Polysaccharide by the Depolymerase in SEC-HPLC

To demonstrate the CPS-degrading activity of the recombinant depolymerase, we analyzed the cleavage activity of the depolymerase against K64 polysaccharide using size exclusion high-performance liquid chromatography (SEC-HPLC). Basically, the purified CPS was dissolved in 50 mM Na_2_HPO_4_ (pH 7.0) to a final concentration of 0.5 mg/mL and incubated with K64-ORF41 (10 μg/mL) or SUMO (10 μg/mL) at 37 °C for 30 min. After incubation, the reactions were terminated by heating at 100 °C for 5 min. The reaction mixture was then analyzed by an HPLC system (Waters, Milford, MA, USA) equipped with a TSKgel G5000 PWxl column (inner diameter 7.8 mm × 300 mm) (Tosoh Corporation Bioscience, Tokyo, Japan) and a refractive index detector (Waters, Milford, MA, USA). The column was run with the mobile phase of the sodium phosphate buffer (pH 7.0) at 1 mL/min and 30 °C.

### 4.10. Alcian Blue Staining

To demonstrate the CPS-degrading activity of the recombinant protein, we conducted a depolymerization assay by Alcian blue staining as previously described [7]. The purified CPS was dissolved in 50 mM Na_2_HPO_4_ (pH 7.0) to final concentration mg/mL and incubated with K64-ORF41 (10 μg/mL) or SUMO (10 μg/mL) at 25 °C for 3 h. After incubation, the reactions were terminated by heating at 100 °C for 5 min. Subsequently, samples were separated by a 10% SDS-PAGE. After electrophoresis, the gel was washed for three times with the fix/wash buffer (25% ethanol, 10% acetic acid in water), then stained by 0.1% Alcian blue (Sigma-Aldrich, Shanghai, China) dissolved in the fix/wash buffer for 15 min in the dark, and destained overnight in the fix/wash buffer.

### 4.11. Capsular Serotyping

The capsular serotypes of these clinical isolates were determined using the Quellung reaction and the agglutination assay as described previously [50,51,52]. Strains were cultured in LB broth at 37 °C overnight followed by centrifugation. The harvested bacteria were then resuspended in 0.5% phenolized saline. A drop of suspension was then mixed with K64-antisera and the methylene blue solution on a glass slide and left for 15 min. A coverslip was applied and the inoculum was observed under the light microscope for the Quellung reaction. The agglutination of bacteria with the K64-antiserum, a method for the rapid identification, was carried out on glass slides. Briefly, strains were diluted to an optical density at 600 nm of 0.6 with phosphate-buffered saline (PBS). An aliquot (20 μL, 3 × 10^6^ to 6 × 10^6^ CFU/mL)) was mixed with K64-antiserum (7.5 μg in 20 μL) and shaken lightly with hands for 1 min to observe the agglutination by eyes.

### 4.12. Sequence Analysis of the cps Region of K. pneumoniae

The genome of *K pneumoniae* strain 6199 was sequenced using Illumina NextSeq (Illumina, San Diego, CA, USA). The sequencing library was prepared using the Illumina Nextera XT kit. The obtained reads were trimmed by Trimmomatic v0.39 [53] and the genome sequence was assembled using Velvet [54]. The *cps* region of *K. pneumoniae* strain 6199 was compared with the K64 reference strain *K. pneumoniae* ATCC-13882 using the CLC Main Workbench version 20 software (https://digitalinsights.qiagen.com; Qiagen, Hilden, Germany). Seven *K pneumoniae* clinical strains, including strain 6821, 6082, 6483, 6938, 6957, 6990 and 8206, were directly amplified by primers K64-*wcaJ*-F (5′-GACAAGTGGTTAAAATTTCAGG-3′) and K64-*wcaJ*-R (5′-TAAAGCATGCACATTCTGAACA-3′), which are specific for the *wcaJ* gene of the *cps* region. All amplified sequences were sequenced and compared with the K64 reference strain, separately. Identification of ISs was performed by ISfinder (http://www-is.biotoul.fr). The sequence of the *cps* locus of the strain 6199 was deposited in GenBank under accession number MW411035 and the *wcaJ* sequences of seven strains, as indicated above, were also submitted to GenBank under accessions: MW432526 for strain 6821, MW432527 for strain 6082, MW432528 for strain 6483, MW432529 for strain 6938, MW432530 for strain 6957, MW432531 for strain 6990, MW432532 for strain 8206.

### 4.13. Effects of pH and Temperature on K64-ORF41 Activity and Stability

The depolymerization activity of K64-ORF41 at different pH levels (3.0–9.0) and at different temperature (25–90 °C) was evaluated by measuring the yield of reducing sugars according to the method reported by Miller et al. [55]. Briefly, the CPS extracted from *K. pneumoniae* strain 2410 was dissolved in the appropriate buffer to a final concentration of 0.5 mg/mL and incubated with K64-ORF41 (10 μg/mL) for 30 min at different pH or temperature. The effect of pH on the enzyme activity was evaluated at 25 °C using the following buffers: 50 mM sodium acetate buffer (pH 3.0–5.0), 50 mM Na_2_HPO_4_ (pH 6.0–7.0), 50 mM Tris-HCl buffer (pH 8.0–9.0) and 100 mM NaHCO_3_ (pH 10.0–11.0). To assess the effect of temperature, assays were performed at optimal pH ranging from 25 °C to 90 °C. The enzyme reaction was terminated by heating at 100 °C for 15 min. Glucose products after the enzymatic reaction were evaluated by using the dinitrosalicylic acid (DNS) method. Briefly, 1.5 mL of the DNS reagent was added to a 500 µL volume of the enzyme-treated products and then heated at 100 °C for 5 min. After cooling with ddH_2_O to room temperature, the optical density at 550 nm was measured. Results were expressed as relative percentage activity. To determine the stability under the different conditions, the K64-ORF41 protein was preincubated at various temperatures or pH conditions for 30 min, and the enzyme activity was assessed and calculated as described above.

### 4.14. Serum Resistance Assay

The serum resistance assay was carried out as described previously [56]. In brief, 2.5 × 10^4^ CFU of strain Y8 in log-phase were centrifuged, resuspended in PBS, and then pretreated for 1 h at 37 °C with K64-ORF41 or SUMO protein at a final concentration of 100 µg/mL. Subsequently, enzyme-treated bacteria were incubated with 75% and 25% active or heat inactivated either heat inactivated or normal human serum from healthy volunteers. After the samples were incubated for 1 h, 2 h, and 3 h at 37 °C, the bacteria were counted in appropriate dilutions on LB agar plates and the survival ratio was calculated.

### 4.15. Neutrophil Killing Assay

The neutrophil killing assay was performed according to the method [57]. Briefly, the HL-60 cell line was differentiated using a standard method [58]. An inoculum containing 1 × 10^5^ CFU of bacteria was pre-incubated with K64-ORF41 or SUMO protein at a final concentration of 100 µg/mL for 1 h at 37 °C. Then, the bacteria were opsonized with 5% normal human serum at room temperature and were incubated with 1 × 10^7^ HL-60 cells. The assay mixture was rotated gently on a horizontal rotator shaker at 37 °C for 30 min. In the end, the mixture was chilled on ice for 20 min to stop the reaction. Each sample was plated onto LB agar plates to calculate colonies. The percentage survival rate of the depolymerase-treated bacteria was determined based on viable counts compared to the untreated control.

### 4.16. Preparation of K64-CPS Antiserum

The antiserum for K64-CPS was generated by Shanghai Ruizhou Biotech Co. In brief, the *K. pneumoniae* K64 polysaccharide was purified from the supernatant of the fermentation culture after centrifugation, precipitation, and chromatography. The purified polysaccharide was then oxidized and coupled to a carry protein. The conjugate was then formulated with adjuvants for the immunization in the rabbit. After immunization, the antiserum from the rabbit was collected and frozen at −80 °C until use. Serum from rabbits immunized with K64-CPS was designated as K64-antisera. Control-serum was also collected from healthy rabbits.

### 4.17. Generation of an Isogenic wcaJ Deletion Mutant in K. pneumoniae

*K. pneumoniae* strain 2410 was used as the wild type *Klebsiella* strain. The 2410-Δ*wcaJ* mutant was conducted by λ Red recombinase and flippase (Flp) recombinase system according to the recombination methods [59,60]. In brief, the *wcaJ* gene was replaced by a flippase recognition target (FRT)-flanked hygromycin resistance gene using *λ* red recombination, and then the resistance gene was eliminated by Flp-FRT recombination. The desired mutant was verified by PCR and Sanger sequencing. PCR primers are described in Supplementary Table S2.

### 4.18. Cloning of wcaJ and Expression in K. pneumoniae

The *wcaJ* gene from *K. pneumoniae* strain 2410 was cloned with specific primers listed in Supplementary Table S2 into pBAD33 vector via the *xba* I and *Hind* III restriction sites. The recombinant plasmid was transformed into the 2410-Δ*wcaJ* mutant and the expression of *wcaJ* was induced with 50 mM l-arabinose. Induced overnight cultures were used in the Quellung reaction, agglutination assay, and the single-spot assay.

### 4.19. Statistics

GraphPad Prism 6 was used for the statistical analysis. The experimental data were tabulated as mean ± SD. In the bivariate analysis, the *t*-test for independent variables was used to analyze normally distributed data, and one-way ANOVA was used to analyze multiple groups. *p* < 0.05 was considered statistically significant.

### 4.20. Ethics Statement

After the patient’s written and informed consent, strain specimens were collected. The experiments and the procedures involved in the current work have been approved by the Ethics Committee of Shanghai Jiao Tong University School of Medicine.

## 5. Conclusions

In this study, we identified and characterized a novel phage-borne depolymerase for K64 capsular polysaccharide (K64-ORF41). Our findings showed that K64-ORF41 exhibited superior activity and specificity to K64-capsulated *K. pneumoniae* and promoted the host immune system in combating the bacterial infection. Our studies supported the potential application of this depolymerase in capsular typing and in the control of CRKP infections.

## 6. Patents

We have applied for a patent about this work. The patent number is 202010910803.6.

## Figures and Tables

**Table 1:** The lytic spectrum of SH-KP152410 and K64-ORF41.

Isolate No.	Genotype (*wzi*)	SH-KP152410		K64-ORF41	Quellung Test	Agglutination
		Plaques	Halos			
13,882 ^1^, 2410 ^2^, Y8 ^2^, 5085, 6003, 6088,6089, 6233, 6353, 6368, 6603, 6682, 6718, 6810, 6954, 6974, 7323, 7366, 7761,7804, 7871, 7880, 7888, 7901, 7982, 8004, 8046, 8047, 8061, 8171, 8207, 8213, 8310, 8315, 8328, 8417, 8422, 8456, 8497, 8608	KL64	+	+	+	+	+
6082, 6199, 6483, 6821, 6938, 6938, 6990, 8206	KL64	−	−	−	−	−
6118, 6876, 7168	KL47	−	−	−	−	−
5146, 5170	KL2	−	−	−	−	−
5080, 5174	KL1	−	−	−	−	−
7830, 6409	KL24	−	−	−	−	−
6795, 7072	KL60	−	−	−	−	−
8191, 8666	KL54	−	−	−	−	−
7607, 7616	KL61	−	−	−	−	−
7485, 6422, 7321, 7485	KL19	−	−	−	−	−

The capsular genotypes of *K. pneumoniae* clinical isolates were identified by wzi genotyping method and the capsular serotypes of these strains were determined by Quellung tests and agglutination assays using K64-antisera (−, negative reaction; +, positive reaction). The spectrum of phage and K64-ORF41 were tested on 67 K. pneumoniae strains using the spot method (−, no lysis; +, showed plaques or halos). ^1^: Serotype K64 reference strain purchased from the ATCC; ^2^: Isolated from patients at the Huashan Hospital, affiliated with Fudan University in Shanghai, China; Besides, other strains were isolated from patients at Renji hospital in 2019 in Shanghai, China, and the capsular genotype of these strains have been reported by *wzi* sequencing in a previous research [34].

## Data Availability

Data available in a publicly accessible repository.

## Supplementary Materials

The following are available online at https://www.mdpi.com/2079-6382/10/2/144/s1, Figure S1: Serotyping by Quellung reaction tests. *K. pneumoniae* strain ATCC 13882, *K. pneumoniae* strain 2410 and *K. pneumoniae* K47 strain 6118 were incubated with K64-antisera or control-serum as described in Methods, Table S1: The location of IS elements in the *wcaJ* region of KL64^+^/K64^−^ strains, Table S2: The primers used for cloning and mutant generation, Table S3: Activity spectrum of K64-ORF41 and results of serological typing.

## References

[1] Fung C.-P., Chang F.-Y., Lee S.-C., Hu B.-S., Kuo B.I.-T., Liu C.-Y., Ho M., Siu L.K. (2002). A global emerging disease of Klebsiella pneumoniae liver abscess: Is serotype K1 an important factor for complicated endophthalmitis?. Gut.

[2] Hidron A.I., Edwards J.R., Patel J., Horan T.C., Sievert D.M., Pollock D.A., Fridkin S.K. (2008). NHSN annual update: Antimicrobial-resistant pathogens associated with healthcare-associated infections: Annual summary of data reported to the National Healthcare Safety Network at the Centers for Disease Control and Prevention, 2006–2007. Infect. Control Hosp. Epidemiol..

[3] Reinert R.R., Low D.E., Rossi F., Zhang X., Wattal C., Dowzicky M.J. (2007). Antimicrobial susceptibility among organisms from the Asia/Pacific Rim, Europe and Latin and North America collected as part of TEST and the in vitro activity of tigecycline. J. Antimicrob. Chemother..

[4] Paczosa M.K., Mecsas J. (2016). Klebsiella pneumoniae: Going on the Offense with a Strong Defense. Microbiol. Mol. Biol. Rev..

[5] Hung C.-H., Kuo C.-F., Wang C.-H., Wu C.-M., Tsao N. (2011). Experimental Phage Therapy in TreatingKlebsiella pneumoniae-Mediated Liver Abscesses and Bacteremia in Mice. Antimicrob. Agents Chemother..

[6] Saylor C., Dadachova E., Casadevall A. (2009). Monoclonal antibody-based therapies for microbial diseases. Vaccine.

[7] Pan Y.J., Lin T.L., Chen Y.H., Hsu C.R., Hsieh P.F., Wu M.C., Wang J.T. (2013). Capsular types of Klebsiella pneumoniae revisited by wzc sequencing. PLoS ONE.

[8] Edwards P.R., Fife M.A. (1952). Capsule Types of Klebsiella. J. Infect. Dis..

[9] Podschun R., Ullmann U. (1998). Klebsiella spp. as Nosocomial Pathogens: Epidemiology, Taxonomy, Typing Methods, and Pathogenicity Factors. Clin. Microbiol. Rev..

[10] Pan Y.J., Lin T.L., Lin Y.T., Su P.A., Chen C.T., Hsieh P.F., Hsu C.R., Chen C.C., Hsieh Y.C., Wang J.T. (2015). Identification of capsular types in carbapenem-resistant Klebsiella pneumoniae strains by wzc sequencing and implications for capsule depolymerase treatment. Antimicrob. Agents Chemother..

[11] Pan Y.J., Fang H.C., Yang H.C., Lin T.L., Hsieh P.F., Tsai F.C., Keynan Y., Wang J.T. (2008). Capsular polysaccharide synthesis regions in Klebsiella pneu-moniae serotype K57 and a new capsular serotype. J. Clin. Microbiol..

[12] Brisse S., Passet V., Haugaard A.B., Babosan A., Kassis-Chikhani N., Struve C., Decré D. (2013). wzi Gene Sequencing, a Rapid Method for Determination of Capsular Type for Klebsiella Strains. J. Clin. Microbiol..

[13] Wyres K.L., Wick R.R., Gorrie C., Jenney A., Follador R., Thomson N.R., Holt K.E. (2016). Identification of Klebsiella capsule synthesis loci from whole genome data. Microb. Genom..

[14] Chuang Y., Fang C., Lai S., Chang S., Wang J. (2006). Genetic Determinants of Capsular Serotype K1 ofKlebsiella pneumoniaeCausing Primary Pyogenic Liver Abscess. J. Infect. Dis..

[15] Wang C., Li P., Niu W., Yuan X., Liu H., Huang Y., An X., Fan H., Zhangxiang L., Mi L. (2019). Protective and therapeutic application of the depolymerase derived from a novel KN1 genotype of Klebsiella pneumoniae bacteriophage in mice. Res. Microbiol..

[16] Pan Y.J., Lin T.L., Chen Y.Y., Lai P.H., Tsai Y.T., Hsu C.R., Hsieh P.F., Lin Y.T., Wang J.T. (2019). Identification of three podoviruses infecting Klebsiella encoding capsule depolymerases that digest specific capsular types. Microb. Biotechnol..

[17] Hsieh P.-F., Lin H.-H., Lin T.-L., Chen Y.-Y., Wang J.-T. (2017). Two T7-like Bacteriophages, K5-2 and K5-4, Each Encodes Two Capsule Depolymerases: Isolation and Functional Characterization. Sci. Rep..

[18] Follador R., Heinz E., Wyres K.L., Ellington M.J., Kowarik M., Holt K.E., Thomson N.R. (2016). The diversity of Klebsiella pneumoniae surface polysaccharides. Microb. Genom..

[19] Majkowska-Skrobek G., Latka A., Berisio R., Squeglia F., Maciejewska B., Briers Y., Drulis-Kawa Z. (2018). Phage-Borne Depolymerases Decrease Klebsiella pneumoniae Resistance to Innate Defense Mechanisms. Front. Microbiol..

[20] Bellich B., Ravenscroft N., Rizzo R., Lagatolla C., D’Andrea M.M., Rossolini G.M., Cescutti P. (2019). Structure of the capsular polysaccharide of the KPC-2-producing Klebsiella pneumoniae strain KK207-2 and assignment of the glycosyltransferases functions. Int. J. Biol. Macromol..

[21] Heidary M., Nasiri M.J., Dabiri H., Tarashi S. (2018). Prevalence of Drug-resistant Klebsiella pneumoniae in Iran: A Review Article. Iran. J. Public Health.

[22] Li B., Zhao Y., Liu C., Chen Z., Zhou D. (2014). Molecular pathogenesis of Klebsiella pneumoniae. Future Microbiol..

[23] Hsu C.R., Lin T.L., Pan Y.J., Hsieh P.F., Wang J.T. (2013). Isolation of a bacteriophage specific for a new capsular type of Klebsiella pneu-moniae and characterization of its polysaccharide depolymerase. PLoS ONE.

[24] Majkowska-Skrobek G., Łątka A., Berisio R., Maciejewska B., Squeglia F., Romano M., Lavigne R., Struve C., Drulis-Kawa Z. (2016). Capsule-Targeting Depolymerase, Derived from Klebsiella KP36 Phage, as a Tool for the Development of Anti-Virulent Strategy. Viruses.

[25] Chaturongakul S., Ounjai P. (2014). Phage-host interplay: Examples from tailed phages and Gram-negative bacterial pathogens. Front. Microbiol..

[26] Maszewska A. (2015). Phage associated polysaccharide depolymerases—characteristics and application. Postepy Hig. Med. Dosw. (Online).

[27] Lin T.-L., Hsieh P.-F., Huang Y.-T., Lee W.-C., Tsai Y.-T., Su P.-A., Pan Y.-J., Hsu C.-R., Wu M.-C., Wang J.-T. (2014). Isolation of a Bacteriophage and Its Depolymerase Specific for K1 Capsule of Klebsiella pneumoniae: Implication in Typing and Treatment. J. Infect. Dis..

[28] Teng T., Li Q., Liu Z., Li X., Liu Z., Liu H., Liu F., Xie L., Wang H., Zhang L. (2019). Characterization and genome analysis of novel Klebsiella phage Henu1 with lytic activity against clinical strains of Klebsiella pneumoniae. Arch. Virol..

[29] Bowers J.R., Kitchel B., Driebe E.M., MacCannell D.R., Roe C., Lemmer D., De Man T., Rasheed J.K., Engelthaler D.M., Keim P. (2015). Genomic Analysis of the Emergence and Rapid Global Dissemination of the Clonal Group 258 Klebsiella pneumoniae Pandemic. PLoS ONE.

[30] Koh T.H., Cao D., Shan Q.Y., Bacon A., Hsu L.Y., Ooi E.E. (2013). Acquired carbapenemases in Enterobactericeae in Singapore, 1996–2012. Pathology.

[31] Huang Y.H., Chou S.H., Liang S.W., Ni C.E., Lin Y.T., Huang Y.W., Yang T.C. (2018). Emergence of an XDR and carbapenemase-producing hypervirulent Klebsiella pneumoniae strain in Taiwan. J. Antimicrob. Chemother..

[32] Cryz S.J., Mortimer P.M., Mansfield V., Germanier R. (1986). Seroepidemiology of Klebsiella bacteremic isolates and implications for vaccine development. J. Clin. Microbiol..

[33] Zhou K., Xiao T., David S., Wang Q., Zhou Y., Guo L., Aanensen D., Holt K.E., Thomson N.R., Grundmann H. (2020). Novel Subclone of Carbapenem-Resistant Klebsiella pneumoniae Sequence Type 11 with Enhanced Virulence and Transmissibility, China. Emerg. Infect. Dis..

[34] Zhang Z.Y., Qin R., Lu Y.H., Shen J., Zhang S., Wang C.Y., Yang Y.Q., Hu F.P., He P. (2020). Capsular polysaccharide and lipopolysaccharide O type analysis of Klebsiella pneumoniae isolates by genotype in China. Epidemiol. Infect..

[35] Kassa T., Chhibber S. (2012). Thermal treatment of the bacteriophage lysate of Klebsiella pneumoniae B5055 as a step for the purifi-cation of capsular depolymerase enzyme. J. Virol. Methods.

[36] Majkowska-Skrobek G., Maciejewska B. (2015). Bacteriophages and Phage-Derived Proteins—Application Approaches. Curr. Med. Chem..

[37] Shu H.-Y., Fung C.-P., Liu Y.-M., Wu K.-M., Chen Y.-T., Li L.-H., Liu T.-T., Kirby R., Tsai S.-F. (2009). Genetic diversity of capsular polysaccharide biosynthesis in Klebsiella pneumoniae clinical isolates. Microbiology.

[38] Zhang W., Guo Y., Li J., Zhang Y., Yang Y., Dong N., Zhu D., He P., Hu F. (2018). In vitro and in vivo bactericidal activity of ceftazidime-avibactam against Carbapenemase–producing Klebsiella pneumoniae. Antimicrob. Resist. Infect. Control..

[39] Lin Y.-T., Jeng Y.-Y., Chen T.-L., Fung C.-P. (2010). Bacteremic community-acquired pneumonia due to Klebsiella pneumoniae: Clinical and microbiological characteristics in Taiwan, 2001–2008. BMC Infect. Dis..

[40] Kaczmarek F.M., Dib-Hajj F., Shang W., Gootz T.D. (2006). High-level carbapenem resistance in a Klebsiella pneumoniae clinical isolate is due to the combination of blaACT-1 β-lactamase production, porin OmpK35/36 insertional inactivation, and down-regulation of the phosphate transport porin PhoE. Antimicrob. Agents Chemother..

[41] Squeglia F., Maciejewska B., Łątka A., Ruggiero A., Briers Y., Drulis-Kawa Z., Berisio R. (2020). Structural and Functional Studies of a Klebsiella Phage Capsule Depolymerase Tailspike: Mechanistic Insights into Capsular Degradation. Structure.

[42] Ernst C.M., Braxton J.R., Rodriguez-Osorio C.A., Zagieboylo A.P., Li L., Pironti A., Manson A.L., Nair A.V., Benson M., Cummins K. (2020). Adaptive evolution of virulence and per-sistence in carbapenem-resistant Klebsiella pneumoniae. Nat. Med..

[43] Venturini C., Ben Zakour N.L., Bowring B., Morales S., Cole R., Kovach Z., Branston S., Kettle E., Thomson N., Iredell J. (2020). Fine capsule variation affects bacteriophage susceptibility in Klebsiella pneumoniae ST258. FASEB J..

[44] Kropinski A.M., Mazzocco A., Waddell T.E., Lingohr E., Johnson R.P. (2009). Enumeration of Bacteriophages by Double Agar Overlay Plaque Assay. Methods Mol. Biol..

[45] Ramos J.-L., Duque E., Huertas M.J., Haïdour A. (1995). Isolation and expansion of the catabolic potential of a Pseudomonas putida strain able to grow in the presence of high concentrations of aromatic hydrocarbons. J. Bacteriol..

[46] Luo R., Liu B., Xie Y., Li Z., Huang W., Yuan J., He G., Chen Y., Pan Q., Liu Y. (2015). Erratum: SOAPdenovo2: An empirically improved memory-efficient short-read de novo assembler. GigaScience.

[47] Delcher A.L., Harmon D., Kasif S., White O., Salzberg S.L. (1999). Improved microbial gene identification with GLIMMER. Nucleic Acids Res..

[48] Söding J., Biegert A., Lupas A.N. (2005). The HHpred interactive server for protein homology detection and structure prediction. Nucleic Acids Res..

[49] Bales P.M., Renke E.M., May S.L., Shen Y., Nelson D.C. (2013). Purification and Characterization of Biofilm-Associated EPS Exopolysac-charides from ESKAPE Organisms and Other Pathogens. PLoS ONE.

[50] Rennie R.P., Duncan I.B.R. (1974). Combined biochemical and serological typing of clinical isolates of Klebsiella. Appl. Microbiol..

[51] Matsen J.M., Blazevic D.J. (1969). Characterization of ornithine decarboxylase-positive, nonmotile strains of the Klebsiella-Enterobacter group. Appl. Microbiol..

[52] Wu M.F., Yang C.Y., Lin T.L., Wang J.T., Yang F.L., Wu S.H., Hu B.S., Chou T.Y., Tsai M.D., Lin C.H. (2009). Humoral immunity against capsule polysaccharide protects the host from magA+ Klebsiella pneumoniae-induced lethal disease by evading Toll-like receptor 4 signaling. Infect. Immun..

[53] Bolger A.M., Lohse M., Usadel B. (2014). Trimmomatic: A flexible trimmer for Illumina sequence data. Bioinformatics.

[54] Zerbino D.R., Birney E. (2008). Velvet: Algorithms for de novo short read assembly using de Bruijn graphs. Genome Res..

[55] Miller G.L. (1959). Use of Dinitrosalicylic Acid Reagent for Determination of Reducing Sugar. Anal. Chem..

[56] Fang C.-T., Chuang Y.-P., Shun C.-T., Chang S.-C., Wang J.-T. (2004). A Novel Virulence Gene in Klebsiella pneumoniae Strains Causing Primary Liver Abscess and Septic Metastatic Complications. J. Exp. Med..

[57] Kobayashi S.D., Porter A.R., Dorward D.W., Brinkworth A.J., Chen L., Kreiswirth B.N., DeLeo F.R. (2016). Phagocytosis and Killing of Car-bapenem-Resistant ST258 Klebsiella pneumoniae by Human Neutrophils. J. Infect. Dis..

[58] Nauseef W.M. (2007). Isolation of human neutrophils from venous blood. Methods Mol. Biol..

[59] Sukhija K., Pyne M., Ali S., Orr V., Abedi D., Moo-Young M., Chou C.P. (2011). Developing an Extended Genomic Engineering Approach Based on Recombineering to Knock-in Heterologous Genes to Escherichia coli Genome. Mol. Biotechnol..

[60] Bi D., Jiang X., Sheng Z.K., Ngmenterebo D., Tai C., Wang M., Deng Z., Rajakumar K., Ou H.Y. (2015). Mapping the resistance-associated mobilome of a car-bapenem-resistant Klebsiella pneumoniae strain reveals insights into factors shaping these regions and facilitates generation of a ‘resistance-disarmed’ model organism. J. Antimicrob. Chemother..

