# Supplementary material for: Identification of a Depolymerase Specific for K64-Serotype Klebsiella pneumoniae: Potential Applications in Capsular Typing and Treatment
Source: Antibiotics (Basel). 2021 Feb 1;10(2):144. doi: 10.3390/antibiotics10020144 (PMC7912862; doi:10.3390/antibiotics10020144)
Supplement: Supplementary file 1 [file antibiotics-10-00144-s001.pdf]

Article

Identification of A Depolymerase Specific for K64-serotype *Klebsiella pneumoniae:* Potential Applications in Capsular Typing and Treatment

Jiayin Li [1,†], Yueying Sheng [2,†], Ruijing Ma [3], Mengsha Xu [1], Fuli Liu [1], Rong Qin [1], Mingxi Zhu [1], Xianchao Zhu [3,*] and Ping He[1,*]

[1] Department of Medical Immunology and Microbiology, Shanghai Jiao Tong University School of Medicine, Shanghai 200025, China; jiayinlee@sjtu.edu.cn (J.L.); xums5812@enzemed.com (M.X.); 2111807117@zjut.edu.cn (F.L.); ashinqr@sjtu.edu.cn (R.Q.); simpsons@sjtu.edu.cn (M.Z.)

[2] College of Medical Technology, Shanghai University of Medicine & Health Sciences, Shanghai 201318, China.; shengyy@sumhs.edu.cn

[3] Shanghai Ruizhou Biotech co. Ltd, Pudong New District, Shanghai 201203, China; rjma@ruizhoubio.com

* Correspondence: jzhu@ruizhoubio.com (X.Z.); hpatsh@sjtu.edu.cn(P.H.)

† These authors contribute equally to this work.

Supplementary Materials:

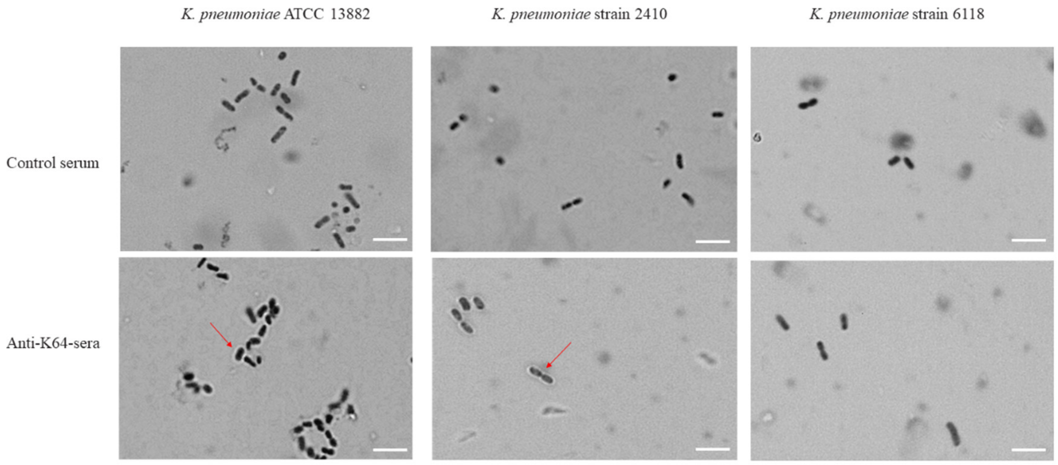

Figure S1. Serotyping by Quellung reaction tests. *K. pneumoniae* strain ATCC 13882, *K. pneumoniae* strain 2410 and *K. pneumoniae* K47 strain 6118 were incubated with K64-antisera or control-serum as described in Methods. Compared with the control, the positive results showed capsular swelling under the light microscope with an oil immersion lens (magnification, 100). Scale bar, 1 μm.

Table S1. The location of IS elements in the *wcaJ* region of $KL64^+$/ $K64^-$ strains.

Isolate no.	Insertion Sequence	Gene	Position of the IS Element* (From the First Nucleotide of *wcaJ*)
6199	IS*Kpn26*	*wcaJ*	685bp
6821	IS*Kpn26*	*wcaJ*	685bp
6082	IS*Kpn26*	*wcaJ*	1018bp
6483	IS*Kpn26*	*wcaJ*	685bp
6938	IS*Kpn26*	*wcaJ*	552bp

6957	IS*Kpn26*	*wcaJ*	34bp
6990	IS*Kpn26*	*wcaJ*	685bp
8206	IS*Kpn26*	*wcaJ*	409bp

*, IS element: IS*5* family transposase IS*Kpn26*.

Table S2. The primers used for cloning and mutant generation.

Primer name	Sequence (5′–3′)
2410-F1-pSUMO3	CAGCAGCAGACGGGAGGATCCGGTGATGGCAGTGAAATAGG
2410-R1-pSUMO3	AGAGCGCTTTTGAAGCTAATTAGAAATCTCACCATAACCATGG
2410-F2-pSUMO3	CCATGGTTATGGTGAGATTTCTAATTAGCTTCAAAAGCGCTCT
2410-R2-pSUMO3	CGGATATATAGCAGATCGTACCGGTACCAATTGGGGATCTT
2410-F3-pSUMO3	AAGATCCCCAATTGGTACCGGTACGATCTGCTATATATCCG
2410-R3-pSUMO3	CTCGAGTGCGGCCGCAAGCTTTCTTTTCACGGGAGCGGTTG
2410--F1	GGTGATGGCAGTGAAATAGG
2410--R1	TCTTTTCACGGGAGCGGTTG
2410-*wcaJ*-check-F	CTCCTGTAGACGTAGAACGA
2410-*wcaJ*-check-R	AGGCTTAAGGGAATCGATGG
pBAD33-*wcaJ*-f	GGTACCCGGGGATCCTCTAGAATGAAAACTTTCACGCATCGTG
pBAD33-*wcaJ*-r	ATCCGCCAAAACAGCCAAGCTTTCAATATGCAGACTTATTAATAAACC
pBAD33-check-f	CATAAGATTAGCGGATCCTACC
pBAD33-check-r	CTACTGCCGCCAGGCAAAT

Table S3. Activity spectrum of K64-ORF41 and results of serological typing.

Isolate no.	K64-ORF41	Quellung test	Agglutination
2410	+	+	+
2410Δ*wcaJ*	-	-	-
2410Δ*wcaJ*-pBAD33-*wcaJ*	+	+	+
6199	-	-	-
6199-pBAD33-*wcaJ*	+	+	+
6821	-	-	-
6821-pBAD33-*wcaJ*	+	+	+
